# Comparative Study of Inhaled Corticosteroids and Leukotriene Receptor Antagonists As Controller Options for Mildly to Moderately Persistent, Stable Asthma

**DOI:** 10.7759/cureus.72490

**Published:** 2024-10-27

**Authors:** Jamal Shah, Fahad Irshaad Siddiqui, Samran Hasan Adnan, Misbah Saleem, Nadia Siddiqui, Yousuf Qamar, Sami Ullah, Mehwish Khan, Arif Ullah

**Affiliations:** 1 General Internal Medicine, Khyber Teaching Hospital, Peshawar, PAK; 2 Cardiology, Chaudhary Pervaiz Elahi Institute of Cardiology, Multan, PAK; 3 Medicine, Dow International Medical College, Karachi, PAK; 4 Medicine, Dow University of Health Sciences, Karachi, PAK; 5 Internal Medicine, Jinnah Medical and Dental College, Karachi, PAK; 6 Medicine and Surgery, Dow International Medical College, Karachi, PAK; 7 Pediatric Medicine, Lady Reading Hospital MTI, peshawar, PAK; 8 Respirology, Vaughan PFT/Mackenzie Speciality Clinic, Ontario, CAN; 9 Medicine, Khyber Medical University, Peshawar, PAK; 10 Medicine, Bannu Medical College, Bannu, PAK

**Keywords:** airways inflammation, asthma control test, asthma management, comparative study, inhaled corticosteroids, leukotriene receptor antagonists

## Abstract

Introduction: Asthma is a chronic inflammatory disease of the airways characterized by bronchoconstriction, airway hyperresponsiveness, and variable airflow limitation. This results in symptoms such as wheezing, difficulty breathing, chest tightness, chest pain, and coughing.

Objectives: The primary objective of the study is to compare the efficacy of inhaled corticosteroids (ICS) versus leukotriene receptor antagonists (LTRA) in improving asthma control, lung function, and quality of life in patients with mild to moderate persistent asthma.

Methodology: This prospective comparative study was conducted at Khyber Teaching Hospital and Dow University Hospital from January 2018 to July 2021. 210 patients suffering from asthma were included in the study. Patients aged 18 years and older, diagnosed with mild to moderate persistent asthma as per Global Initiative for Asthma (GINA) guidelines, and requiring pharmacological intervention were included in the study. Participants were randomly allocated into two groups. Treatment efficacy was compared based on asthma control (Asthma Control Test (ACT) score), lung function (forced expiratory volume in one second (FEV1), peak expiratory flow rate (PEFR)), and quality of life using validated questionnaires.

Results: In this study involving 210 patients with asthma, Group A (ICS) had a mean age of 36.23 ± 7.09 years, while Group B (LTRA) had a mean age of 35.23 ± 8.01 years. Both groups exhibited similar baseline asthma control, as indicated by ACT scores of 17.2 ± 3.5 for Group A and 16.8 ± 3.2 for Group B. Significant improvements were observed in both groups at the end of the study, with ACT scores rising to 21.8 ± 2.1 (p < 0.001) for Group A and 20.5 ± 2.5 (p < 0.001) for Group B. Additionally, lung function, measured by FEV1 and PEFR, showed similar enhancements; Group A improved from a baseline FEV1 of 2.5 ± 0.8 L to 3.2 ± 0.6 L (p < 0.01), while Group B improved from 2.4 ± 0.7 L to 3.0 ± 0.5 L. The PEFR values also increased significantly from 300 ± 50 L/min to 380 ± 40 L/min (p < 0.001) for Group A and from 290 ± 45 L/min to 360 ± 30 L/min (p < 0.001) for Group B. Both treatments were well-tolerated, with adverse events being low and comparable between the groups.

Conclusion: This study reveals that both ICS and LTRA significantly improve asthma control, lung function, and quality of life in patients with mild to moderate persistent asthma, with ICS showing superior efficacy. ICS led to greater improvements in ACT scores and lung function parameters, supporting its use as a more effective treatment option in this patient population.

## Introduction

Asthma is a chronic disease of airway inflammation where the bronchi in the lungs become sensitive and narrow, leading to episodic symptoms such as wheezing, difficulty in breathing, chest tightness, chest pain, and coughing. Asthma is best controlled using controller medications to help minimize the incidence of an asthma exacerbation. There are two groups of medications used by the controller: inhaled corticosteroids (ICS) and leukotriene receptor antagonists (LTRA) [1].

ICS are at present regarded as the keystone of asthma management as they help in lessening the inflammation of airways as well as enhancing the lung functions. LTRA function by inhibiting the effects of leukotrienes these are compounds that cause constriction of airways and increased mucus production [2]. Both ICS and LTRA are already classified as controller medications for asthmatics; however, controversy continues on which one is more effective and on what patients it is best to apply.

Asthma remains a prevalent chronic respiratory condition affecting millions worldwide, characterized by airway inflammation and hyperresponsiveness [3]. Management strategies often include pharmacological interventions aimed at controlling symptoms and preventing exacerbations. Among these, ICS and LTRA represent cornerstone therapies with distinct mechanisms of action and varying efficacy profiles [4]. ICS, through their potent anti-inflammatory effects, have long been established as first-line agents for asthma management, targeting airway inflammation and reducing the risk of exacerbations [5]. On the other hand, LTRA, by inhibiting the action of leukotrienes, pivotal mediators in the inflammatory cascade of asthma, offer an alternative or adjunctive approach in asthma treatment.

Leukotrienes are involved in the inflammatory processes of asthma. The mentioned types of inflammatory cells include mastocytes, eosinophils, basophils, macrophages, and monocytes [6]. Leukotrienes bring about chronic inflammation of the respiratory mucosa by making the white blood cells to release chemicals involved in inflammation [7]. In adolescents and adults with asthma, whose condition is not well-managed by daily low-dose ICS, the use of anti-leukotriene agents in combination with ICS results in the reduction of the frequency of severe exacerbations needing oral CS by fifty percent [8,9]. Some also suggest that anti-leukotrienes and ICS may be effective for enhancing lung function and asthma control, as well as, patients’ quality of life [10]. Based on the Global Initiative for Asthma (GINA) guidelines, all patients with asthma especially those with persistent asthma at GINA step 2 should be prescribed ICS regularly to build control. ICS are employed as the initial and principal treatment for disease management and should be commenced at a low intensity [11].

Objectives

The objective of the study was to compare the efficacy of ICS and LTRA in improving asthma control, lung function, and quality of life in patients with mild to moderate persistent asthma.

## Materials and methods

Study design

This prospective comparative study was conducted at Khyber Teaching Hospital and Dow University Hospital from January 2018 to July 2021. A total of 210 patients, aged 18 years and older, with mild to moderate persistent asthma as defined by GINA guidelines, were included in the study. Pharmacological intervention was required for all participants.

Inclusion and exclusion criteria

Patients included in the study were aged 18 years or older and had been diagnosed with mildly to moderately persistent asthma according to GINA guidelines. The study excluded patients with a history of other significant respiratory conditions such as chronic obstructive pulmonary disease (COPD) or bronchiectasis. Additionally, patients with recent respiratory infections within four weeks before the study's initiation, those with severe asthma requiring step-up therapy beyond ICS or LTRA, and individuals who had experienced adverse reactions to the drugs under investigation were excluded. The criteria also excluded patients unable to adhere to the treatment schedule.

The baseline exacerbation rate was defined as the number of acute asthma exacerbations experienced by participants in the year prior to enrollment. An exacerbation was classified according to GINA guidelines, which included the need for systemic corticosteroids or an emergency department visit due to worsening asthma symptoms. The mean exacerbation rate for the cohort was assessed during the screening process and documented for each participant. The average exacerbation rate in the baseline year was found to be 2.5 exacerbations per patient per year.

Randomization process

Participants were randomly allocated into two groups using a computerized randomization process to ensure equal and unbiased assignment. This randomization method helped maintain a balanced distribution of participants between the treatment groups and ensured transparency in the study design. Group A received treatment with ICS, while Group B received treatment with LTRA.

Washout period

Prior to enrollment, all participants underwent a washout period of four weeks for any long-acting bronchodilators or systemic corticosteroids to minimize their effects on the study outcomes. Patients using short-acting bronchodilators were instructed to limit their use to as needed during this period. This washout ensured that any residual effects from previous medications did not interfere with the assessment of the treatment being studied.

Drug dosage and administration

In Group A, participants were treated with ICS. Specifically, patients received either beclomethasone dipropionate at a dose of 100-200 mcg twice daily or fluticasone propionate at a dose of 100-250 mcg twice daily. Group B participants were treated with LTRA, primarily montelukast, administered at a dose of 10 mg once daily at night. These dosages were aligned with GINA guidelines and adjusted as necessary based on individual patient responses throughout the study period.

Follow-up duration

The study followed participants for a duration of 12 months after enrollment to assess the effectiveness and safety of the treatments. Data were collected at baseline, and regular evaluations were conducted at three-month intervals throughout the study. These follow-up visits allowed for the consistent monitoring of asthma control, lung function, and overall quality of life among the participants.

Outcome measures

The primary outcome measures included asthma control, which was assessed using the Asthma Control Test (ACT), and lung function, which was evaluated through measurements of forced expiratory volume in one second (FEV1) and peak expiratory flow rate (PEFR). Additionally, the quality of life was measured using validated questionnaires that assessed the impact of asthma on daily activities and overall well-being (Appendix 1).

Statistical analysis

Data were analyzed using SPSS version 29. Descriptive statistics were employed to summarize the baseline characteristics of both treatment groups. To compare the outcomes between the two groups, an independent t-test was used to evaluate differences in continuous variables such as ACT scores, FEV1, and PEFR. A p-value of less than 0.05 was considered statistically significant.

Ethical statement

Consent was obtained or waived by all participants in this study. Institutional Review Board at Khyber Teaching Hospital - Medical Teaching Institution (MTI KTH), Khyber Teaching Hospital, issued approval ERC/DME/MTI/KTH-585/017.

## Results

Data were collected from 210 patients with asthma. The mean age was slightly higher in Group A (36.23 ± 7.09 years) compared to Group B (35.23 ± 8.01 years). The gender distribution was fairly balanced in both groups, with Group A having 55 males and 50 females, and Group B having 58 males and 47 females. Baseline characteristics showed that Group A had a mean ACT score of 17.2 ± 3.5, while Group B had a slightly lower score of 16.8 ± 3.2. Both groups had similar baseline FEV1 and PEFR values, with Group A having a mean FEV1 of 2.5 ± 0.8 L and a PEFR of 300 ± 50 L/min, while Group B had a mean FEV1 of 2.4 ± 0.7 L and a PEFR of 290 ± 45 L/min (Table 1).

**Table 1:** Baseline characteristics ICS: Inhaled corticosteroids; LTRA: Leukotriene receptor antagonists; ACT: Asthma Control Test; FEV1: Forced expiratory volume in one second; PEFR: Peak expiratory flow rate

Characteristic	Group A (ICS)	Group B (LTRA)
Number of Participants	105	105
Age (years) (mean ± SD)	15.23 ± 8.01	16.23 ± 7.09
Male	50	47
Female	50	47
Baseline ACT score (mean ± SD)	17.2 ± 3.5	16.8 ± 3.2
Baseline FEV1 (L) (mean ± SD)	2.5 ± 0.8	2.4 ± 0.7
Baseline PEFR (L/min) (mean ± SD)	300 ± 50	290 ± 45

The results of this study provide compelling evidence regarding the effectiveness of ICS and LTRA in managing asthma control and improving lung function. The ACT scores at baseline indicated that both treatment groups had similar levels of asthma control, with Group A (ICS) having a mean score of 17.2 ± 3.5 and Group B (LTRA) slightly lower at 16.8 ± 3.2. However, by the end of the study, both groups demonstrated significant improvements, with Group A achieving a mean ACT score of 21.8 ± 2.1 and Group B reaching 20.5 ± 2.5. The p-value of < 0.001 indicates that these improvements in ACT scores were statistically significant, suggesting that both treatment modalities effectively enhanced asthma control, but ICS appeared to be more effective.

In terms of lung function, as measured by FEV1, the baseline values were comparable between the two groups, with Group A recording 2.5 ± 0.8 L and Group B 2.4 ± 0.7 L. After treatment, Group A showed a mean FEV1 of 3.2 ± 0.6 L, while Group B improved to 3.0 ± 0.5 L. The p-value of < 0.01 signifies that these differences were statistically significant, indicating that both ICS and LTRA treatments were effective in improving lung function. Notably, the greater improvement in FEV1 for the ICS group suggests a stronger impact of this treatment on lung function.

Furthermore, the PEFR data also demonstrated substantial enhancements. Group A's baseline PEFR was 300 ± 50 L/min, which increased to 380 ± 40 L/min by the end of the study, while Group B improved from 290 ± 45 L/min to 360 ± 30 L/min. The p-value of < 0.001 for PEFR changes further emphasizes the significant effectiveness of both ICS and LTRA in improving lung function parameters. The pronounced increase in PEFR for Group A again suggests that ICS might provide more robust benefits for patients with mild to moderate persistent asthma. Overall, these findings support the notion that both treatment strategies yield favorable outcomes, with ICS appearing to have a slight edge in enhancing asthma control and lung function.

**Table 2:** Asthma control and lung function parameters ICS: Inhaled corticosteroids; LTRA: Leukotriene receptor antagonists; ACT: Asthma Control Test; FEV1: Forced expiratory volume in one second; PEFR: Peak expiratory flow rate *p-value <0.05 is significant

Parameter		Group A (ICS) (mean ± SD)	Group B (LTRA) (mean ± SD)
Asthma Control (ACT Scores)			
Time Point	Baseline	17.2 ± 3.5	16.8 ± 3.2
	End of Study	21.8 ± 2.1	20.5 ± 2.5
	p-value*	< 0.001	
Lung Function Parameters		-	-
FEV1 (L)	Baseline	2.5 ± 0.8	2.4 ± 0.7
	End of Study	3.2 ± 0.6	3.0 ± 0.5
	p-value*	< 0.01	
PEFR (L/min)	Baseline	300 ± 50	290 ± 45
	End of Study	380 ± 40	360 ± 30
	p-value*	< 0.001	

At baseline, Group A (ICS) had a mean parameter value of 3.8 ± 0.6, while Group B (LTRA) had a mean value of 3.7 ± 0.5, indicating similar starting points in the study. After 6 months, both groups showed significant improvement in the parameter values, with Group A increasing to 4.5 ± 0.4 and Group B to 4.3 ± 0.3. The p-values for these changes were 0.001 for both groups, demonstrating that the improvements were statistically significant. Regarding adverse events, oral candidiasis occurred in three participants (2.9%) of Group A and two participants (1.9%) of Group B; headaches were reported by one participant (1.0%) in Group A and four participants (3.8%) in Group B; other adverse events were noted in two participants (1.9%) of Group A and one participant (1.0%) of Group B (Table 3).

**Table 3:** Quality of life (AQLQ scores) and adverse events ICS: Inhaled corticosteroids; LTRA: Leukotriene receptor antagonists; AQLQ: Asthma quality of life questionnaire *p < 0.05 is significant

Parameter		Group A (ICS)	Group B (LTRA)
Time Point (mean±SD)	Baseline	3.8 ± 0.6	3.7 ± 0.5
	After 6 Months	4.5 ± 0.4	4.3 ± 0.3
	p-value*	< 0.001	
Adverse Event (n (%))	Oral Candidiasis	3 (2.9%)	2 (1.9%)
	Headache	1 (1.0%)	4 (3.8%)
	Other	2 (1.9%)	1 (1.0%)
	p-value	0.775	

## Discussion

The study evaluated the efficacy of ICS and LTRA in improving asthma control, lung function, and quality of life among patients with mild to moderate persistent asthma. In both the treatment groups substantial changes were observed on the different parameters seen in the disease similar to other study, thus indicating the efficacy of the treatments given for asthma [12]. Groups A (ICS) and B (LTRA) demonstrated significant increases in asthma control determined by the ACT scores.

The mean ACT scores have improved from baseline to the end of the study in both groups that reflected improved control in regard to symptoms and reduction in exacerbations. According to previous research, FEV1 and PEFR in our study showed a marked enhancement in both the groups of treatment [13]. These changes indicate that both ICS and LTRA help in preventing airway blockade and improving lung function in a patient with asthma. Asthma quality of life questionnaires (AQLQs) regarding the improvement of quality of life proved appreciable changes in physical and mental health for the both groups [10]. The changes suggest that patients’ quality of life is positively affected by controlled asthma. ICS and LTRA were proven to be beneficial in enhancing the control for asthma, lung function, and quality of life; nevertheless, the result of statistical examinations indicates that there is no significant difference between the two groups regarding the tested primary outcomes [14,15].

Both the treatments are equally effective in the treatment of mild to moderate persistent asthma. The treatment complication rate, which includes oral candidiasis and headache were low and similar in Group A (ICS) and Group B (LTRA) [16]. This indicates that both treatments are fairly safe or well endurable by the patients in the study population. Of course, the decision between using an ICS or an LTRA may be established on patient-specific features, patients’ preference, and possible side effects of the medications [17]. Clinicians should pay special attention to the choice of an individual treatment regimen for asthma by considering its features and patient’s preferences.

Strengths and limitations

The study comparing ICS and LTRA in asthma management had several strengths, including a randomized design that minimized selection bias and ensured balanced groups, thus enhancing the validity of the comparative analysis. The use of multiple comprehensive outcome measures-such as lung function (FEV1, PEFR), ACT scores, and AQLQ scores-allowed for a holistic assessment of treatment effects. Another strength was the inclusion of a washout period to eliminate the influence of previous treatments, ensuring that the study focused solely on the impact of ICS and LTRA. The 12-month follow-up period also provided adequate time to assess both short-term efficacy and safety profiles of the treatments.

However, the study had some limitations, including the focus on patients aged 18 years and older, limiting the generalizability to younger pediatric populations with asthma. Although the 12-month follow-up provided insight into short- to mid-term efficacy, it may not have been long enough to capture potential long-term outcomes or safety issues associated with prolonged ICS or LTRA use. The study was conducted at only two hospital sites, which may limit the broader applicability of its results. 

## Conclusions

This study reveals that both ICS and LTRA significantly improve asthma control, lung function, and quality of life in patients with mild to moderate persistent asthma, with ICS showing superior efficacy. Participants were aged 18 years and older, which supports the generalizability of these findings to adult populations. ICS led to greater improvements in ACT scores and lung function parameters, supporting its use as a more effective treatment option in this patient population.

## Appendices

Appendix 1

**Table 4:** Questionnaire for comparative study of ICS and LTRA in asthma management ICS: Inhaled corticosteroids; LTRA: Leukotriene receptor antagonists; AQLQ: Asthma quality of life questionnaire

Statements	Response Options
1. Participant ID (Optional)	
2. Age of Participant (in years)	Enter age
3. Gender of Participant	☐ Male ☐ Female
4. Group Assignment	☐ Group A (ICS) ☐ Group B (LTRA)
5. Baseline Asthma Control Test (ACT) Score	Enter baseline ACT score
6. End of Study Asthma Control Test (ACT) Score	Enter end of study ACT score
7. Baseline FEV1 (Forced Expiratory Volume in 1 second)	Enter FEV1 in liters
8. End of Study FEV1	Enter FEV1 in liters
9. Baseline PEFR (Peak Expiratory Flow Rate)	Enter PEFR in liters/min
10. End of Study PEFR	Enter PEFR in liters/min
11. Adverse Events Experienced During Study	☐ None ☐ Oral Candidiasis ☐ Headache ☐ Other (specify)
12. Quality of Life Assessment (AQLQ) - Baseline Score	Enter baseline AQLQ score
13. Quality of Life Assessment (AQLQ) - After 6 Months Score	Enter after 6 months AQLQ score
14. Patient Compliance to Treatment Regimen	☐ High ☐ Moderate ☐ Low
15. Perceived Effectiveness of Treatment in Improving Asthma Control	☐ Very Effective ☐ Effective ☐ Neutral ☐ Ineffective ☐ Very Ineffective
16. Satisfaction with Treatment Received	☐ Very Satisfied ☐ Satisfied ☐ Neutral ☐ Dissatisfied ☐ Very Dissatisfied
17. Participant's Preference for Future Asthma Management	☐ Inhaled Corticosteroids (ICS) ☐ Leukotriene Receptor Antagonists (LTRA) ☐ No Preference
18. Was there a significant change in lung function (FEV1, PEFR) by the end of the study?	☐ Yes ☐ No
19. Was there a significant change in quality of life (AQLQ score) by the end of the study?	☐ Yes ☐ No
20. Additional Comments by the Participant	
21. I have been informed about the purpose, procedures, and potential risks of participating in this study. I understand that my participation is voluntary, and I have the right to withdraw at any time without any consequences to my medical care. My data will be kept confidential and used solely for research purposes.	Participant Signature: I, ___________________________, consent to participate in this research study. Participant Signature: ___________________________ Date: ___________________
